# Myc is involved in Genistein protecting against LPS-induced myocarditis *in vitro* through mediating MAPK/JNK signaling pathway

**DOI:** 10.1042/BSR20194472

**Published:** 2020-06-18

**Authors:** Chunhua Huang, Yan Zhang, Hongli Qi, Xintan Xu, Lin Yang, Jianjun Wang

**Affiliations:** 1 Department of Pediatrics, The Affiliated Hospital of Jining Medical University, No. 89 Guhuai Road, Jining City, Shandong Province, P.R. China; 2 Department of 5 Internal Medicine, Qihe County People’s Hospital, No. 118, Qiyan Street, Qihe County, Dezhou City, Shandong Province, P.R. China; 3 Department of Cardiology, The Affiliated Hospital of Jining Medical University, No. 89 Guhuai Road, Jining City, Shandong Province, P.R. China

**Keywords:** apoptosis, Genistein, ILs, Myc, TNF-α

## Abstract

**Background**: Genistein is widely used as a pharmacological compound as well as a food additive. However, the pharmaceutical effects of Genistein on myocarditis and its potential mechanisms have not been studied in detail. **Methods**: H9c2 cells were continuously stimulated by lipopolysaccharide (LPS) for 12 h to simulate the *in vitro* model of myocarditis injury. DrugBank, String, and GEO dataset were used to investigate specific genes that interacting with Genistein. KEGG and GO enrichment analysis were employed to explore Myc-related signaling pathways. Biological behaviors of H9c2 cells were observed with the support of cell counting kit-8, MTT and flow cytometry. Expression levels of cytokines including TNF-α and ILs were evaluated by enzyme-linked immunosorbent assay. Western blot was applied to detect the expression of Myc and MAPK pathway related proteins. **Results**: Genistein alleviated the damage of H9c2 cells subjected to LPS from the perspective of elevating cells growth ability, and inhibiting cells apoptosis and inflammatory response. Through bioinformatics analysis, we identified Myc as the potential target of Genistein in myocarditis, and MAPK as the signaling pathway. Significantly, Myc was highly up-regulated in myocarditis samples. More importantly, by performing biological experiments, we discovered that Genistein relieved H9c2 cells apoptosis and inflammatory reaction which caused by LPS stimulation through inhibiting Myc expression. Additionally, the marked augmentation of p-P38 MAPK and p-JNK expression in LPS-induced cardiomyocyte model were blocked by Genistein and si-Myc. **Conclusions**: Our research revealed that Myc mediated the protective effects of Genistein on H9c2 cells damage caused by LPS partly through modulation of MAPK/JNK signaling pathway.

## Introduction

Myocarditis is a disease featured by the existence of inflammatory infiltrates in cardiac tissue, which is the leading cause of acute cardiac failure, sudden death and dilated cardiomyopathy in children and young people [1–3]. The short-term prognosis of acute myocarditis is generally well, but varies greatly by etiology. Those patients with initial recovery may experience recurrent dilated cardiomyopathy and cardiac failure years later [4]. Additionally, the symptoms of myocarditis are similar to those of acute coronary syndrome, accompanied by chest pain, abnormal electrocardiogram, elevated serum creatine kinase, and hemodynamic instability [5]. Therefore, further study is needed to investigate effective drugs to treat myocarditis and to reveal the molecular mechanisms of myocarditis treatment.

Genistein, the main isoflavone in soybean, is a plant polyphenolic compound following chemical structure analogous to mammalian estrogen [6]. Genistein has various molecular effects covering inhibiting inflammation, promoting apoptosis, and regulating steroid hormone receptors and metabolic pathways [7]. Isoflavones including Genistein have been discovered to perform health protective functions in aging-related and hormone-dependent diseases as well as cardiovascular diseases [8,9]. For example, Genistein has a potential role in the prevention of breast cancer in terms of metabolism [6]. Moreover, Genistein exhibits a broad range of impacts on inflammation and NAD^+^ metabolism via paracrine and endocrine signaling pathways [10]. Additionally, Genistein has a capacity to influence the effects of the pancreatic endocrine system and elevate the sensitivity of insulin receptor [11]. Importantly, Genistein exerts a protective effect in several cardiovascular disease, such as stroke [12], myocardial ischemia/reperfusion injury [13]. Despite numerous health benefits of Genistein, its efficacy and mechanisms in myocarditis have not been adequately estimated. Therefore, it is of great significance to explore the function of Genistein in myocarditis.

By enquiring DrugBank database, MYC proto-oncogene bHLH transcription factor (Myc) was identified as a well target gene of Genistein. Myc, pertaining to the Myc gene family, is a multiple-effect transcription factor that involves in various cellular processes, covering cell growth, apoptosis, metabolism, differentiation, and DNA repair [14]. To date, Myc overexpression continually appeared in small-cell lung cancer, myeloid leukemia, breast and cervix carcinoma [15]. In addition, Myc expression is very high in the heart of newborn mouse for the acceleration of myocardial cell growth and heart evolution [16]. Additionally, Myc family appears to be essential for CVB3-induced myocardial apoptosis [17], and Myc is connected with a mitochondrial-related apoptosis pathway in myocarditis [18]. In the present study, we discovered that Genistein alleviated H9c2 cells damage induced by LPS through deactivating Myc expression and inacting MAPK/JNK signaling pathway.

## Materials and methods

### Data procurement

DrugBank and String database were used to inquire the target proteins related to Genistein. The genes encoding these target proteins were analyzed by KEGG and GO enrichment to investigate related signaling pathways. GEO dataset with accession number GSE35182 (includes six cases with heart CVD3 10 dpi and six cases with heart pbs 10 dpi) were applied to detect differentially expressed genes in myocarditis sample, and Myc expression in myocarditis was analyzed based on the GEO dataset.

### Cell culture and treatment

Rat cardiomyocytes H9c2 cells were acquired from American Type Culture Collection (ATCC), and incubated in Dulbecco’s modified Eagle’s medium (DMEM) supplemented with 10% fetal bovine serum (FBS), 100 U/ml penicillin and 100 μg/ml streptomycin in a humidified incubators at 37°C with 5% CO_2_. The cells injury model of myocarditis was formed by H9c2 cells with lipopolysaccharide (LPS, 10 μg/ml) stimulation for 12 h.

Sequences of si-con and si-Myc provided by Genepharma (Shanghai, China) were transfected into H9c2 cells with the help of Lipofectamine 2000 (Thermo Fisher Scientific, U.S.A.) to regulate the expression of Myc. Genistein was gained from Sigma and dissolved with dimethylsulfoxide (DMSO) to 10 µM. After pretreatment with 10 µM Genistein for 12h, H9c2 cells were stimulated with 10 μg/ml LPS for 12 h. H9c2 cells in the control group were cultured with equal concentration of DMSO.

### Cell proliferation test

Cell counting kit-8 (CCK-8) reagent was applied to assess the proliferation of H9c2 cells. First, the cells were implanted into 96-well plates with the density of 5 × 10^3^ cells/well and incubated overnight. After treating with Genistein, LPS or/and si-Myc, the absorbance of H9c2 cells was calculated at 0, 24, 48, and 72 h. Before calculation, H9c2 cells were incubated with CCK-8 reagent for another 1.5 h under the above conditions. Finally, the absorbance was calculated by a microplate reader at the wavelength of 450 nm.

### MTT assay

H9c2 cells collected in the logarithmic phase were seeded in 96-well plates with the density of 2 × 10^3^ cells/well. Afterwards, 20 μl of MTT reagent (5 g/l, Sigma, Saint Louis, U.S.A.) was loaded into per well daily over 1 3-day time course and cultured for 4 h every day, accompanied by the supplemented of 150 μl of DMSO and cultured for another 15 min. The optical absorbance was assessed at the wavelength of 570 nm.

### Cell apoptosis test

Annexin V/propidium iodide (PI, Invitrogen, U.S.A.) double staining was adopted to estimate H9c2 cells apoptosis. Primarily, the collected cells were centrifuged and re-suspended with 1 × binding buffer. Then, 5 µl of Annexin V/FITC and 10 µl of PI were supplemented into aforesaid buffer and mixed away from the light at approximately 25°C for 10 min. Finally, the flow cytometry (BD Biosciences, Franklin Lakes, NJ, U.S.A.) was used to evaluate the apoptosis rate of H9c2 cells.

### RNA extraction and quantitative real-time PCR (qRT-PCR)

First, whole RNA was acquired from treated cells with the support of Trizol (Takara, Japan). Then, RNA was reverse transcribed into cDNA with the help of PrimeScript™RT reagent kit (Takara, Japan). The Applied Biosystems 7500 Real Time PCR System was applied to execute quantitative experiments under the support of SYBR Premix Ex Taq II (Takara, Japan).

### Western blot

After treatment, H9c2 cells were lysed under the support of RIPA buffer accompanied by protease inhibitor. After isolation by SDS-polyacrylamide gel electrophoresis, the proteins were transferred to polyvinylidene difluoride membranes. Next, after blocking by 5% defatted milk, the membranes were incubated with primary antibodies (Abcam, U.K.) against Myc (ab39688, 1:1000), p-P38 MAPK (ab4822, 1:1000), P38 MAPK (ab31828, 1:1000), p-JNK (ab124956, 1:3000), JNK (ab179461, 1:1000), and GAPDH (ab181602, 1:5000) at 4°C for 24 h. After washing with PBS, the membranes were incubated with secondary antibodies at 25°C for another 2 h. Lastly, the signals of protein bands were developed with enhanced chemiluminescence (ECL) and quantified with ImageJ software.

### Enzyme-linked immunosorbent assay

The expression of inflammatory cytokine TNF-α, IL-6, IL-8 and IL-10 were assessed with the help of enzyme-linked immunosorbent assay (ELISA) kit (Beyotime Biotechnology, China). All the experimental steps were carried out according to the instructions of the supplier.

### Statistical analysis

The values were processed with SPSS22.0 and GraphPad Prism 6.0, which exhibited as mean ± standard deviation (SD). We performed Student’s *t*-test and One-Way ANOVA analysis followed by Bonferroni post hoc test to calculate the differences between quantitative variables. Two-tailed *P* value less than 0.05 was regarded statistically significant.

## Results

### Genistein ameliorates H9c2 cells damage induced by LPS

**Figure 1:**
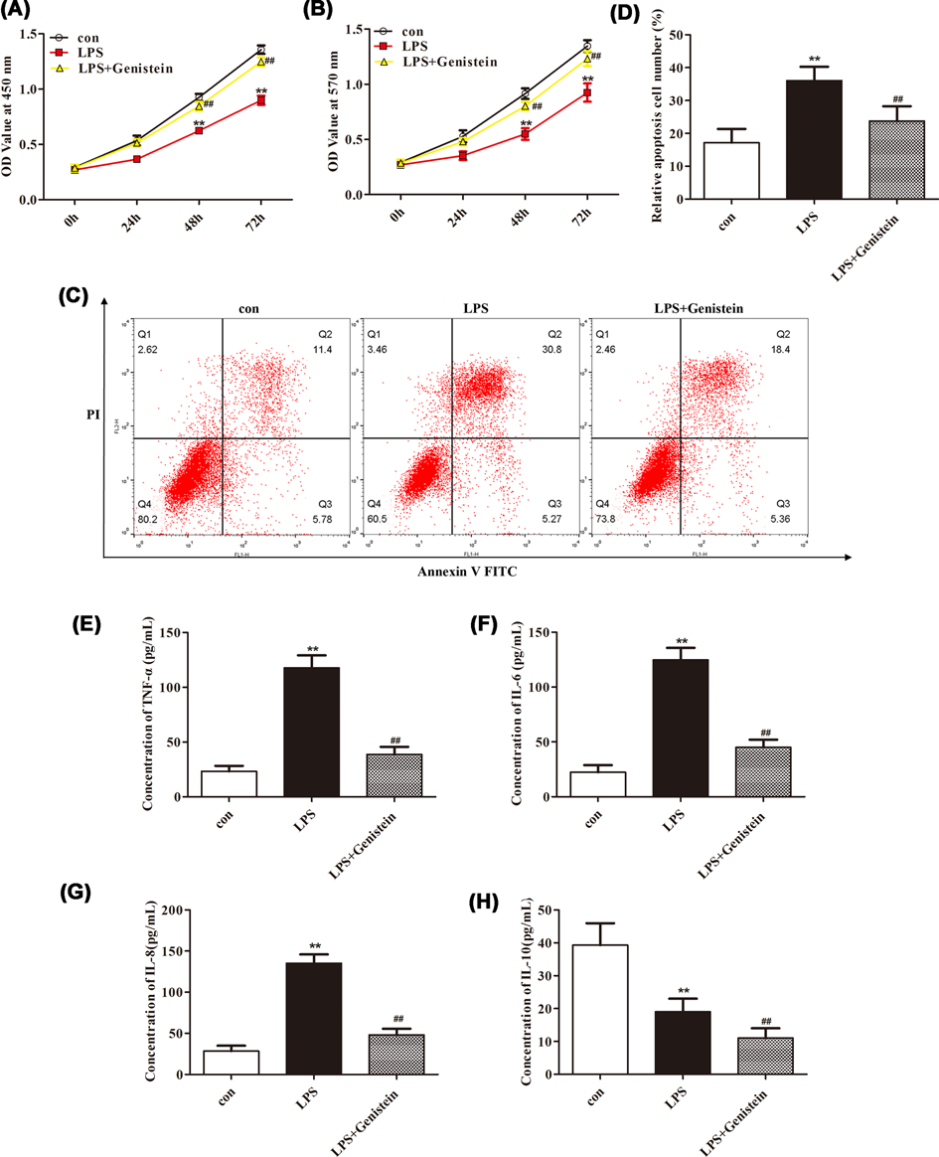
Genistein alleviated LPS-induced H9c2 cells damage by limiting apoptosis and the expression of inflammatory factors (**A,B**) The results from CCK-8 and MTT assays showed that the decreased proliferation of H9c2 cells induced by LPS was attenuated with the help of Genistein. (**C,D**) The increased apoptosis of H9c2 cells caused by LPS was relieved after Genistein treatment. The up-regulation of pro-inflammatory factors including TNF-α (**E**), IL-6 (**F**), and IL-8 (**G**) in H9c2 cells caused by LPS were inhibited under the guidance of Genistein. (**H**) The down-regulation of anti-inflammatory factor IL-10 in H9c2 cells caused by LPS was suppressed with the support of Genistein. **P<0.01 vs. control, ^##^*P*<0.01 vs. LPS.

Considering the numerous benefits of Genistein in metabolic disease and cancers [7], we attempted to investigate the effects of Genistein on myocarditis. After stimulation by LPS, the proliferation of H9c2 cells was significantly decreased with CCK-8 and MTT detection, and the apoptosis and inflammatory response of H9c2 cells were obviously aggravated accompanied by an increase in the number of apoptotic H9c2 cells, a marked augmentation of TNF-α, IL-6, IL-8 expression as well as a significantly reduction of IL-10 expression (Figure 1, *P*<0.01). While, after treatment with Genistein, the adverse reactions of H9c2 cells stimulated by LPS were significantly alleviated and improved as we observed the elevation of proliferation ability and suppression of apoptosis and inflammatory response (Figure 1, *P*<0.01). These phenomena insinuated that Genistein has a potential protective effect in myocarditis.

### Associations among Genistein, Myc, and MAPK signaling are evaluated

**Figure 2:**
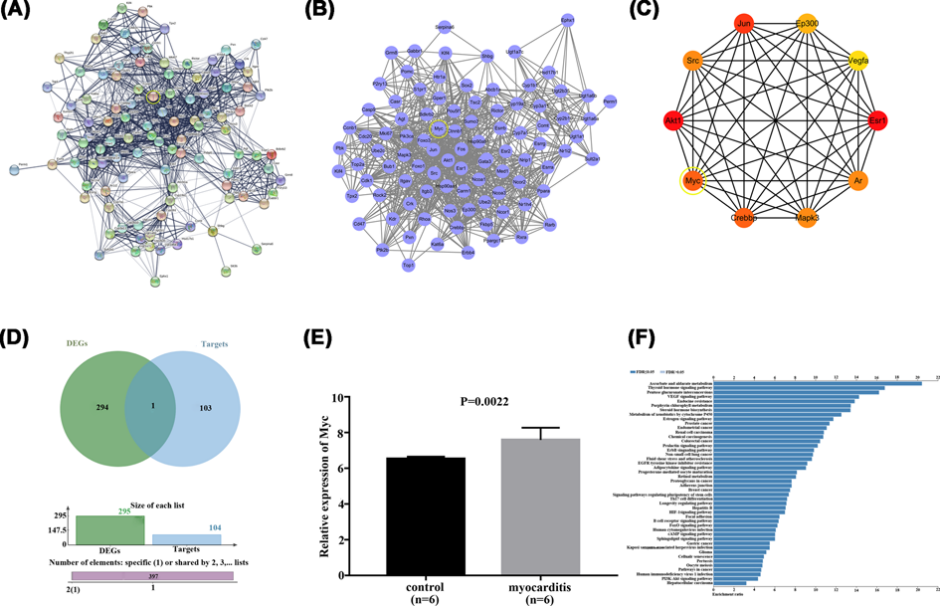
Searching for genes and signaling pathway related to Genistein with the guidance of several bioinformatics prediction websites (**A,B**) The 104 target proteins were obtained with the help of String database. (**C**) The top 10 genes were acquired by sorting the 104 genes encoding these proteins with the support of hubgene analysis. (**D**) Myc gene was acquired by the intersection with the target genes and differential genes. (**E**) The expression of Myc in myocarditis was up-regulated by inquiring the GEO database, *P*=0.0022. (**F**) KEGG pathway enrichment analysis was performed to explore the potential signaling pathway related to Myc.

**Figure 3:**
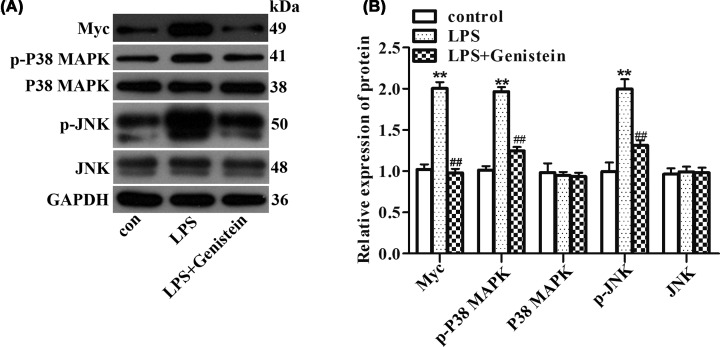
Associations among Genistein, Myc and MAPK pathway were identified by Western blot (**A,B**) The expression of Myc, p-P38 MAPK, P38 MAPK, p-JNK, and JNK were detected with the help of Western blot after different treatment. **P<0.01 vs. control, ^##^*P*<0.01 vs. LPS.

Based on the above results, we sought to find the potential mechanisms of the protective effects of Genistein in myocarditis. By consulting DrugBank database, we obtained 13 target proteins that directly interact with Genistein, including ESR2, TOP2A, PTK2B, NCOA1, ESR1, NCOA2, ESRRB, ESRRA, NR1I2, AKT1, GPER1, CYP1B1, and SHBG. Next, we used the String database to retrieve the proteins that interact with the 13 target proteins, resulting in 91 proteins (Figure 2A,B). Next, the top 10 genes were acquired by sorting the 104 genes encoding these proteins with the support of hubgene analysis (Figure 2C). Then, the genes encoding these 104 proteins were intersected with 305 differentially expressed genes obtained from the analysis of the myocarditis dataset in GEO database to obtain the only gene Myc (Figure 2D). The expression of Myc in myocarditis was highly up-regulated compared with the control (Figure 2E, *P*=0.0022). Additionally, through GO and KEGG pathway enrichment analysis, a close relationship between Myc and MAPK pathway was presented (Figure 2F). Afterwards, Western blot experiment was carried out to excavate the effects of Genistein on Myc and MAPK pathway in H9c2 cells. The data revealed that the proteins expression of Myc, p-P38 MAPK and p-JNK were significantly elevated in H9c2 cells suffered from LPS. However, by Genistein treatment, these proteins expression were reversed (Figure 3A,B, *P*<0.01). These information implied that Genistein functioned in myocarditis might through regulation of Myc and MAPK/JNK signaling pathway.

### Genistein relieves LPS-induced H9c2 cells injury with the support of suppressing Myc and inactivating MAPK/JNK signaling pathway

**Figure 4:**
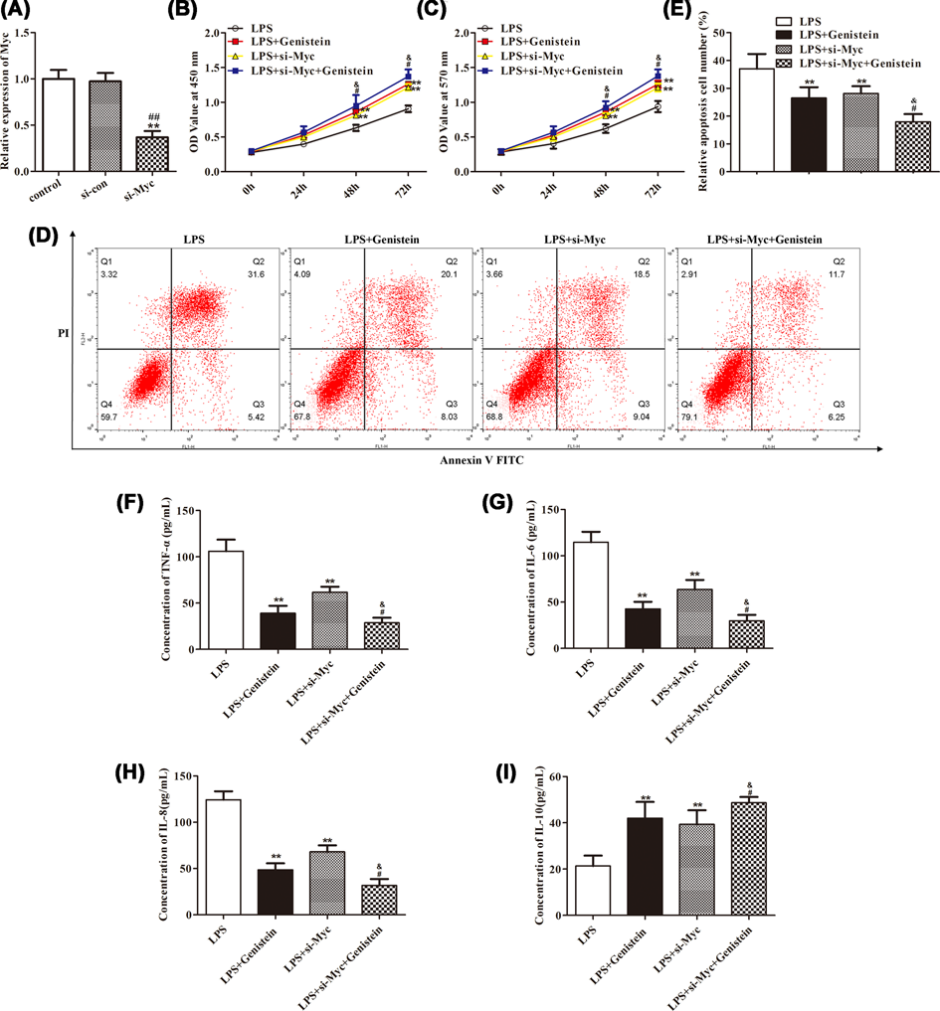
The protective effects of Genistein on H9c2 cells were mediated by Myc (**A**) The interference efficiency of Myc was proved to be successful. The H9c2 cells activity was evaluated by CCK-8 (**B**) and MTT kits (**C**) after different treatment. (**D,E**) The apoptosis of H9c2 cells was assessed by flow cytometry after different treatment. (**F–I**) The inflammatory factors were calculated by ELISA kit after different stimulation. **P<0.01 vs. LPS, ^#^*P*<0.05 vs. LPS+Genistein, ^&^*P*<0.05 vs. LPS+si-Myc.

To identify the above hypothesis, CCK-8, MTT, flow cytometry, and ELISA experiments were implemented. First, the interference of Myc was successful after treatment with si-Myc compared with the si-con group (Figure 4A, *P*<0.01). Next, the results indicated that H9c2 cells activity was increased after treatment with Genistein or si-Myc in LPS condition. While, together treatment with Genistein and si-Myc, the activity of H9c2 cells was slightly elevated compared with only processing with Genistein or si-Myc (Figure 4B,C, *P*<0.05). Similarly, the apoptosis of H9c2 cells was decreased following the procession with Genistein or si-Myc in LPS condition. However, by together stimulation with Genistein and si-Myc, the number of H9c2 cells was mildly reduced in contrast with just treatment with Genistein or si-Myc (Figure 4D,E, *P*<0.05). By Genistein or si-Myc treatment in LPS condition, the expression of pro-inflammatory factors including TNF-α, IL-6 and IL-8 were decreased, while anti-inflammatory factor IL-10 increased. Whilst, together stimulation by Genistein and si-Myc, the expression of TNF-α, IL-6 and IL-8 were gently reduced, while IL-10 was lightly elevated compared with only treatment with Genistein or si-Myc (Figure 4F–I, *P*<0.05). These findings suggested that the relaxation effects of Genistein on H9c2 cells were mediated by Myc.

**Figure 5:**
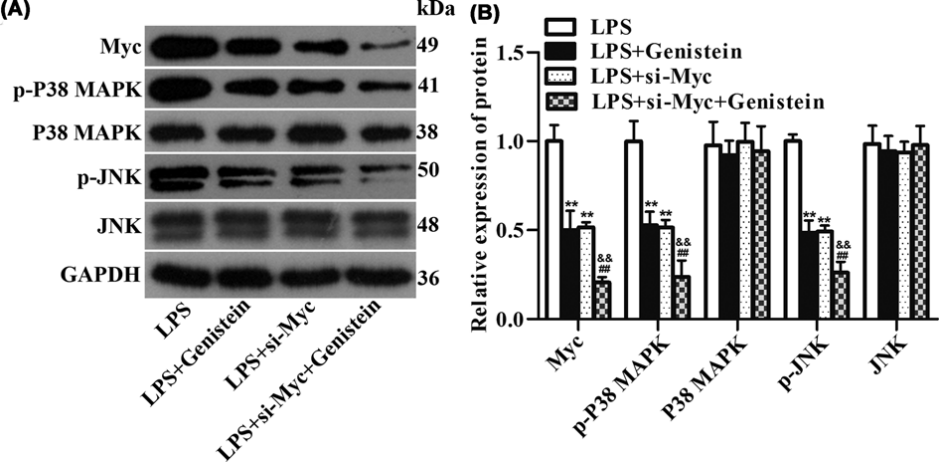
The mitigation effects of Genistein on H9c2 cells were partly regulated by MAPK/JNK pathway (**A,B**) The expression of Myc, p-P38 MAPK, P38 MAPK, p-JNK, and JNK were estimated by Western blot after different stimulation. **P<0.01 vs. LPS, ^##^*P*<0.01 vs. LPS+Genistein, ^&&^*P*<0.01 vs. LPS+si-Myc.

Next, Western blot assay stated that the expression of p-P38 MAPK and p-JNK were significantly decreased after treatment with Genistein or si-Myc. However, the expression of p-P38 MAPK and p-JNK were gently reduced after together stimulation with Genistein and si-Myc compared with only processing with Genistein or si-Myc (Figure 5A,B, *P*<0.01). These observations illustrated that the alleviation of Genistein on H9c2 cells damage was partly mediated by MAPK/JNK signaling pathway.

## Discussion

We presented the potential therapeutic effects of Genistein on LPS-induced myocarditis. Our research drew two major conclusions. First, Genistein attenuated LPS-induced H9c2 cells injury by inhibiting apoptosis and inflammation. Second, the protective effects of Genistein on myocarditis were partly mediated by Myc and MAPK/JNK signaling pathway.

With the further discovery of Genistein function, its medicinal efficacy has been valued by more and more researchers [19,20]. The best-know efficacy of Genistein is as a chemotherapeutic drug for different types of cancer, mainly by changing apoptosis, cell cycle, angiogenesis, and inhibiting metastasis [21]. The efficacy of Genistein in myocarditis has not been fully evaluated. Interestingly, the results from our study clearly revealed that Genistein significantly inhibited the apoptosis and inflammatory reactions of H9c2 cells induced by LPS. By inquiring several database, Myc was found to be a potential target of Genistein. Recently, some basic cellular functions of Myc have been set up. Myc is a major regulator of cell growth and proliferation. In addition, Myc can induce cells to apoptosis, modulate cells senescence, and participate in DNA damage response [22]. Whilst, Myc expression plays a dual role in cells survival and tissue homeostasis [23–25]. For example, an increase level of Myc protein make epithelial cells highly sensitive to pro-apoptosis stimuli such as DNA damage [23]. However, due to the strong mitotic signal, proto-oncogene induced cell senescence requires the down-regulation of Myc expression [25]. Interestingly, in our study, Myc was overexpressed in myocarditis patients and in LPS-induced H9c2 cells, which was in steps with the observations from Xiao et al. [26]. Moreover, the up-regulation of Myc induced by LPS in H9c2 cells can be blocked by Genistein, indicating the potential relation between Genistein and Myc in myocarditis.

Previous study uncovered that Genistein regulates some pathophysiological pathways that are usually deregulated in obesity, metabolic syndrome, and cancer [27,28]. In the present study, we discovered that Genistein can alleviate cells damage by regulating MAPK/JNK signaling pathway. Previous study has revealed that LPS stimulation excites a train of signaling pathways in H9c2 cells, primarily by activating MAPK/JNK signaling pathway, which actuates apoptotic signals to induce apoptosis and generate myocardial injury [29]. There is sufficient evidence that MAPK family plays a conspicuous role in the transcriptional regulation of most inflammatory genes induced by LPS, which are major causes of infectious cardiac dysfunction [30–33]. Luckily, through preliminary data mining, we discovered that Myc has a close relationship with MAPK signaling pathway. More importantly, several studies uncovered that Myc expression was positively related to MAPK signaling pathway in asthma airway remodeling [34], osteoarthritis [35], and some tumors [36,37]. In steps with the previous, we discovered that depletion of Myc in LPS model can block the expression of p-P38 MAPK and p-JNK. Additionally, the expression of p-P38 MAPK and p-JNK were significantly up-regulated in LPS-induced H9c2 cells, which can be limited after treatment with Genistein. These findings insinuated that the remission of H9c2 cells damage by Genistein was partly mediated by MAPK/JNK signaling pathway

It has been indicated that inflammatory reactions are implicated in myocarditis and that a wide range of inflammatory factors, including TNF-α and IL-6, might be toxic to myocardial cells [38]. Given the importance of inflammatory response in myocarditis, we examined the expression of inflammation-related factors. Our study exhibited that pro-inflammation factors including TNF-α, IL-6, and IL-8 were significantly up-regulated and anti-inflammation factor IL-10 was obviously down-regulated in LPS-induced H9c2 cells to promote cells damage. Interestingly, these observations of plasma markers were consistent with the findings in isolated atrial fibrillation, which given more evidence for our results [39]. Whilst, Genistein can alleviate these phenomena by suppression of Myc expression and inactivation MAPK/JNK signaling pathway.

Several shortcomings in the article need to be revealed. First, these findings are based only on cellular models and need to be validated *in vivo*. Second, the indicators we detected are relatively single, so we need to detect a wider range of biological indicators in the future.

Taken together, these observations revealed that Myc monitored the protective effects of Genistein on H9c2 cells against injury from regulating the progress in apoptosis and inflammatory response by regulation of MAPK/JNK signaling pathway.

## Abbreviations

CCK-8: cell counting kit-8
DMSO: dimethylsulfoxide
ELISA: enzyme-linked immunosorbent assay
LPS: lipopolysaccharide
